# Highly Sensitive Detection Method for HV69-70del in SARS-CoV-2 Alpha and Omicron Variants Based on CRISPR/Cas13a

**DOI:** 10.3389/fbioe.2022.831332

**Published:** 2022-04-12

**Authors:** Mengwei Niu, Yao Han, Xue Dong, Lan Yang, Fan Li, Youcui Zhang, Qiang Hu, Xueshan Xia, Hao Li, Yansong Sun

**Affiliations:** ^1^ State Key Laboratory of Pathogen and Biosecurity, Beijing Institute of Microbiology and Epidemiology, Beijing, China; ^2^ Faculty of Life Science and Technology, Kunming University of Science and Technology, Kunming, China

**Keywords:** variants, SARS-CoV-2, CRISPR/Cas13a, nucleic acid detection, lateral flow strip

## Abstract

As SARS-CoV-2 variants continue to evolve, identifying variants with adaptive diagnostic tool is critical to containing the ongoing COVID-19 pandemic. Herein, we establish a highly sensitive and portable on-site detection method for the HV69-70del which exist in SARS-CoV-2 Alpha and Omicron variants using a PCR-based CRISPR/Cas13a detection system (PCR-CRISPR). The specific crRNA (CRISPR RNA) targeting the HV69-70del is screened using the fluorescence-based CRISPR assay, and the sensitivity and specificity of this method are evaluated using diluted nucleic acids of SARS-CoV-2 variants and other pathogens. The results show that the PCR-CRISPR detection method can detect 1 copies/μL SARS-CoV-2 HV69-70del mutant RNA and identify 0.1% of mutant RNA in mixed samples, which is more sensitive than the RT-qPCR based commercial SARS-CoV-2 variants detection kits and sanger sequencing. And it has no cross reactivity with ten other pathogens nucleic acids. Additionally, by combined with our previously developed ERASE (Easy-Readout and Sensitive Enhanced) lateral flow strip suitable for CRISPR detection, we provide a novel diagnosis tool to identify SARS-CoV-2 variants in primary and resource-limited medical institutions without professional and expensive fluorescent detector.

## Introduction

Some of SARS-CoV-2 variants have demonstrated possible immune escape and increased transmissibility, such as the Alpha variant (B.1.1.7) and Omicron variant (B.1.1.529), which have caused worldwide concern (Kirby, 2021). The Alpha variant, firstly identified in southern England, has enhanced binding affinity with human ACE2 receptor and increased viral infectivity (Meng et al., 2021; Ramírez et al., 2021; Yaniv et al., 2021). The Omicron variant, firstly identified in South Africa, has enhanced viral replication and infection ability, making it quickly became the current dominant pandemic strain globally (Karim and Karim, 2021; Dächert et al., 2022). Both of Alpha and Omicron variants contain HV69-70del mutant, which has been shown to have potential biological implications and associated with human immune response evasion (Andrés et al., 2020; Ho et al., 2021). Hence, as global SARS-CoV-2 outbreaks continue, early identification of SARS-CoV-2 variants is critical. To date, two mainly methods have been used to detect SARS-CoV-2 variants. The most commonly used is gene sequencing technology, including Sanger sequencing and next-generation sequencing, and the other detection method commonly employed is based on reverse transcription quantitative PCR (RT-qPCR). While, both of them are time-consuming, depending on professional equipment and gene database, which is not suitable for those resource-limited regions (Khan et al., 2020; Perchetti et al., 2021). Therefore, an urgent need exists for the development of a sensitive, specific and on-site detection method to accurately identify SARS-CoV-2 variants on time.

The nucleic acid detection technology based on clustered regularly interspaced short palindromic repeats (CRISPR)/CRISPR-associated (Cas) system provided the opportunity for researchers to achieve accurate and on-site test (Harrington et al., 2018). Up to now, there are various CRISPR/Cas-systems had been developed for COVID-19 diagnosis such as Cas9, Cas12a/b and Cas13a. Shortly after the COVID-19 pandemic started, Doudna’s group developed a LbuCas13a-based method for the quantitative detection of SARS-CoV-2 RNA without a pre-amplification (Ackerman et al., 2020; Fozouni et al., 2021). Lately, Rauch and colleagues also developed an on-site SARS-CoV-2 diagnosis tool based on CRISPR/Cas13a system and minimal LED infrastructure (Rauch et al., 2021). Additionally, researchers also showed the practicability of CRISPR detection system for identifying SARS-CoV-2 variants (Broughton et al., 2020; Wang R. et al., 2021), including D614G and N501Y variants (Huang et al., 2021; Kumar et al., 2021; Zhang et al., 2021a). For instance, Wang and colleagues developed a light-up RNA aptamer signaling-CRISPR-Cas13 amplification method for SARS-CoV-2 variants D614G identification (Wang Y. et al., 2021). While, the limits of detection (LOD) and specificity of exist detection strategies are still need to improve for SARS-CoV-2 variants identification (Patchsung et al., 2020). In this study, by combining our previously developed PCR-CRISPR genotypic detection (Wang S. et al., 2021) and the easy-readout and sensitive enhanced (ERASE) lateral flow strip (Li H. et al., 2021), we demonstrated a highly sensitive, and specific detection method for the SARS-CoV-2 HV69-70del. And this method facilitated us to identify SARS-CoV-2 variants in primary and resource limited medical institutions without professional and expensive fluorescent detector.

## Methods

### Materials

The SARS-CoV-2 sequence (NC_045512.2) was obtained from the NCBI database. The spike gene of the SARS-CoV-2 RNA reference material was obtained from the China National Institute Metrology (NIM-RM5208). The primers and probes were purchased from Sangon Biotech Co., Ltd. (Shanghai, China). Plasmids containing 144del, 243del, 3675del, L452R, N501Y, P681H, D614G, and other SARS-CoV-2 mutation sites were synthesized by Tianyi Huiyuan Biotech Co., Ltd. In addition, the ten pathogens nucleic acids for be preserved by Academy of Military Medical Sciences.

### Reagents and Instruments

NTP mix (art. No. N0466S), and T7 transcription kit (art. No. E2050S) and T7 RNA polymerase (art. No. E2050S), and RNase inhibitors (art. No. E2050S) were purchased from the New England Biological Laboratory (NEB) of the United States, Cas13a (art. No. db005) protein was purchased from Nanjing Genscript Biotechnology Co., Ltd. The RNaseAlert™ QC System v2; art. No. 4479769 was purchased from Thermo Fisher company of the United States. In addition, a One-step TB Green PrimeScript RT-PCR Kit (art. No. RR066A) was purchased from Takara, Japan, and 2× Super Pfx MasterMix (art. No. CW2965M) was purchased from Jiangsu Cowin Biotechnology Co., Ltd. SARS-CoV-2 (strain B.1.1.7) S gene N501Y and HV69-70del mutation detection kits (art. No. JC10226N) were purchased from Jiangsu BioPerfectus Technologies Co., Ltd. The PCR Instrument Applied Biosystems, Thermo Scientific, USA; fluorescence quantitative PCR instrument of Mastercycler-realplex4 was produced by Eppendorf (Germany).

### Design and Preparation of Primers and crRNAs

RT-PCR primers (Supplementary Table S1) targeting SARS-CoV-2 HV69-70del and N501Y mutations were designed using the Primer-BLAST tool from the NCBI website. To design the specific crRNAs of the SARS-CoV-2 variant, the genome sequence was downloaded and compared from GISAID; crRNAs were designed to nucleic acid fragments of 28 bp. The crRNA transcripts were prepared by annealing with T7-crRNA-F and crRNA-R. crRNAs were prepared by first synthesizing DNA with a T7 promoter sequence. The crRNA DNA was then annealed to a short T7 sequence with the HiScribe T7 Fast High Yield RNA Synthesis Kit (NEB) and incubated overnight with T7 polymerases at 37°C. Finally, the RNA clean XP volume (Beckman Coulter) was used for crRNA purification at a ratio of 1:1.8. All crRNA sequences used in this study are available in Supplementary Table S2.

### RT-PCR Condition of Synthesized RNA Template

RT-PCR was performed using a One-step TB Green PrimeScript RT-PCR Kit, using a standard manufacturer protocol (RR066A, TAKARA, Japan). The reaction system contained 12.5 μL of 2× One-Step TB Green RT-PCR Buffer, 0.5 μL of PrimeScript RT enzyme mix, 2 μL of diluted RNA template, 2.5 U Takara Ex Taq HS, 0.2 μM F and R primer, and DNase/RNase-free water up to 25 μL. The thermal cycling procedure was 42°C for 5 min and 85°C for 10 min, followed by 40 cycles at 95°C for 5 s, 55°C for 30 s, and 72°C for 30 s. After amplification, 5 μL of the RT-PCR product was analyzed using the PCR-CRISPR fluorescence method for 60 min.

### RT-qPCR Quantitative Detection and Mutation Detection of RNA Template

Different SARS-CoV-2 mutation sites were executed using SARS-CoV-2 (strain B.1.1.7) S gene N501Y and HV69-70del mutation detection kits, according to the manufacturer’s protocol (JC10226N, bioPerfectus, Jiangsu). The thermal cycling procedure was 50°C for 10 min and 97°C for 1 min, followed by 45 cycles at 97°C for 5 s, and 58°C for 30 s. All RT-qPCR experiments included quality controls, comprising DNase/RNase-free water instead of RNA template (non-template control, NC), in each run.

### Cas13a-Mediated crRNA/Cas13a Collateral Cleavage

Cas13a-mediated RNA cleavage contain 1.6 IU/μL RNase inhibitor (NEB), 20 mM N-2-hydroxyethylpiperazine-N-2-ethane sulfonic acid (HEPES), 25 nM Cas13a, 2 μM crRNA, 2.5 mM ribonucleoside triphosphates (rNTP) (NEB), 2 nM reporter RNA, 1 IU/μL T7 RNA polymerase (NEB), 10 mM MgCl_2_, and 5 μL of the RT-PCR amplified product. The reaction is carried out at 37°C for 60 min using a fluorescence quantitative PCR system. Alternatively, the reaction system is incubated for 30 min at 37°C, and the reaction mixture is added to the ERASE strip subsequently.

### ERASE Lateral Flow Strip Detection

This experiment used test notes called ERASE lateral flow strips (Li H. et al., 2021) that were previously developed by our research team. The ERASE strip was visualized and analyzed according to the “band-cutting method;” if, on the lateral flow strip, the C-band was visible, while the T-band was absent, a positive result was recorded, whereas if both the T-band and C-band were visible, the result was recorded as negative. If the C-band was not visible on the ERASE strip, the test was deemed to have failed and the lateral flow strip was replaced prior to retesting.

### Statistical Analysis

Statistical significance was determined by unpaired two-tailed Student’s *t*-tests using GraphPad Prism 8.0.2 software. Data are represented as mean ± SEM (ns, no significant difference, *p < 0.05, **p < 0.01, ***p < 0.001, ****p < 0.0001).

## Results

### Identification of Efficient Primers and crRNA for SARS-CoV-2 HV69-70del Detection

**FIGURE 1:**
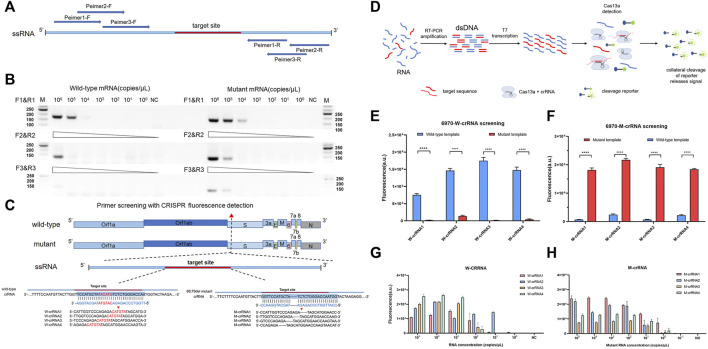
Identifying the sensitivity and specificity of different crRNA and primer sets with RT-PCR followed by Cas13a detection. **(A)** Schematic of the primer design. **(B)** Agarose gel electrophoresis confirms the amplification efficiency of RT-PCR 69-70 primers. **(C)** Schematic of SARS-CoV-2 mutant target regions and the crRNA sequences used for detection. The target site is blue, and the mutant site is red. **(D)** Schematic of RT-PCR + CRISPR detection. **(E)** Highly specificity detection of wild-type and mutant template for the differentiation of SARS-CoV-2 wild-type crRNA targets using Cas13a. **(F)** Highly specificity detection of wild-type and mutant template for the differentiation of SARS-CoV-2 mutant crRNA targets using Cas13a. **(G)** Sensitivity detection of four different wild-type crRNAs carried out using PCR-CRISPR and wild-type templates. **(H)** Sensitivity detection of four different mutant crRNAs carried out using PCR-CRISPR and mutant templates. (*n* = 3 technical replicates, two-tailed Student t-test; **, *p* < 0.01; ****, *p* < 0.0001; ns, no significant difference; bars represent mean ± s.e.m.).

The RT-PCR amplified efficiency is determined by the reverse and forward primer regions, so we designed the corresponding amplification primers in different regions (Figure 1A and Supplementary Table S1) and screened them by agarose gel electrophoresis. The results showed that primer 1 had an obvious amplification band compared to primers 2 and 3, which had an obvious band in the template of 10^4^ copies/μL, while primers 2 and 3 had obvious bands when amplifying the template of 10^5^ copies/μL (Figure 1B). To establish a highly sensitive and specific detection method, we selected four candidate crRNAs targeting the wild-type (W-6970-1, 2, 3, 4) and four candidate crRNAs targeting the mutant-type (M-6970-1, 2, 3, 4) (Figure 1C and Supplementary Table S2) in the HV69-70del region of SARS-CoV-2 genome. Then we used fluorescence-based PCR-CRISPR detection method to screening these crRNA (Figure 1D). The results show that all crRNAs in different locations could distinguish between wild-type RNA templates and HV69-70del templates (Figures 1E,F). To determine the sensitivity of different crRNAs, synthetic RNA templates were prepared using a serial dilution that ranged from 1 × 10^5^ to 1 × 10^−1^ copies/μL in each reaction. The results show that W-crRNA-2 detection sensitivity is higher for detecting wild RNA templates and the detection limit is 10^1^ copies/μL. M-crRNA-1 detection sensitivity is higher for detecting HV69-70del RNA templates and the detection limit is 10^0^ copies/μL (Figures 1G,H). Therefore, W-crRNA-2 and M-crRNA-1 were selected for further studies.

### Sensitivity and Specificity Evaluation of PCR-CRISPR in SARS-CoV-2 HV69-70del Detection

**FIGURE 2:**
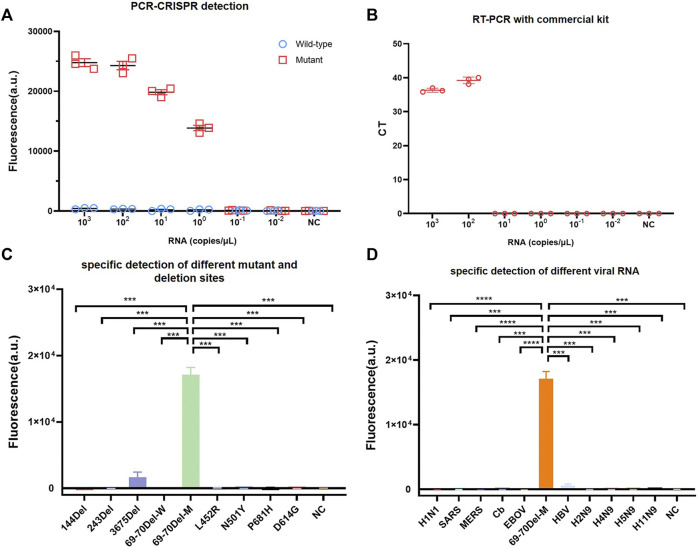
Identifying the sensitivity and specificity of PCR-CRISPR detection and comparing sensitivity with other nucleic acid detection tools. **(A)** Sensitivity detection of wild and mutant templates carried out using PCR-CRISPR and M-crRNA-1. **(B)** Detection analysis of mutant RNA dilution series with RT-qPCR with a commercial kit. **(C)** PCR-CRISPR can discriminate different viral mutant or deletion sites. **(D)** PCR-CRISPR achieves specific detection of different viral RNA. (*n* = 3 technical replicates, two-tailed Student t-test; ***, *p* < 0.001; ****, *p* < 0.0001; bars represent mean ± s.e.m.).

To compare PCR-CRISPR detection with the existing TaqMan probe RT-qPCR, we purchased SARS-CoV-2 (strain B.1.1.7) S gene N501Y and HV69-70del mutation detection kits (BioPerfectus). The results showed that PCR-CRISPR was more sensitive than SARS-CoV-2 (strain B.1.1.7) S gene N501Y and HV69-70del mutation detection kits and can detect 1 × 10^0^ copies/μL template RNA. In contrast, SARS-CoV-2 (strain B.1.1.7) S gene N501Y and HV69-70del mutation detection kits detected only 1 × 10^2^ copies/μL template RNA (Figures 2A,B). In addition, fluorescence signals were observed to increase over time and become stabilized after 30 min (Supplementary Figure S1). Therefore, in all subsequent studies involving combined Cas13a and lateral flow strip, 30 min was set as the Cas13a detection time.

To evaluate the specificity of PCR-CRISPR detection of SARS-CoV-2 HV69-70del, we synthesized a plasmid that contained 144del, 243del, 3675del, L452R, N501Y, P681H, and D614G. We evaluated the specificity of SARS-CoV-2 HV69-70del in different pathogens for nucleic acid detection, and extracted H1N1, SARS, MERS, Cb, EBOV, HBV, H2N9, H4N9, H5N9, and H11N9 nucleic acids for PCR-CRISPR fluorescence detection. The results show that the PCR-CRISPR fluorescence detection method has high specificity for detecting different SARS-CoV-2 mutation sites (Figure 2C) and nucleic acids of different pathogens (Figure 2D).

### The PCR-CRISPR Method Detects the Low-Proportion Mutant Genes in Mixed Samples

**FIGURE 3:**
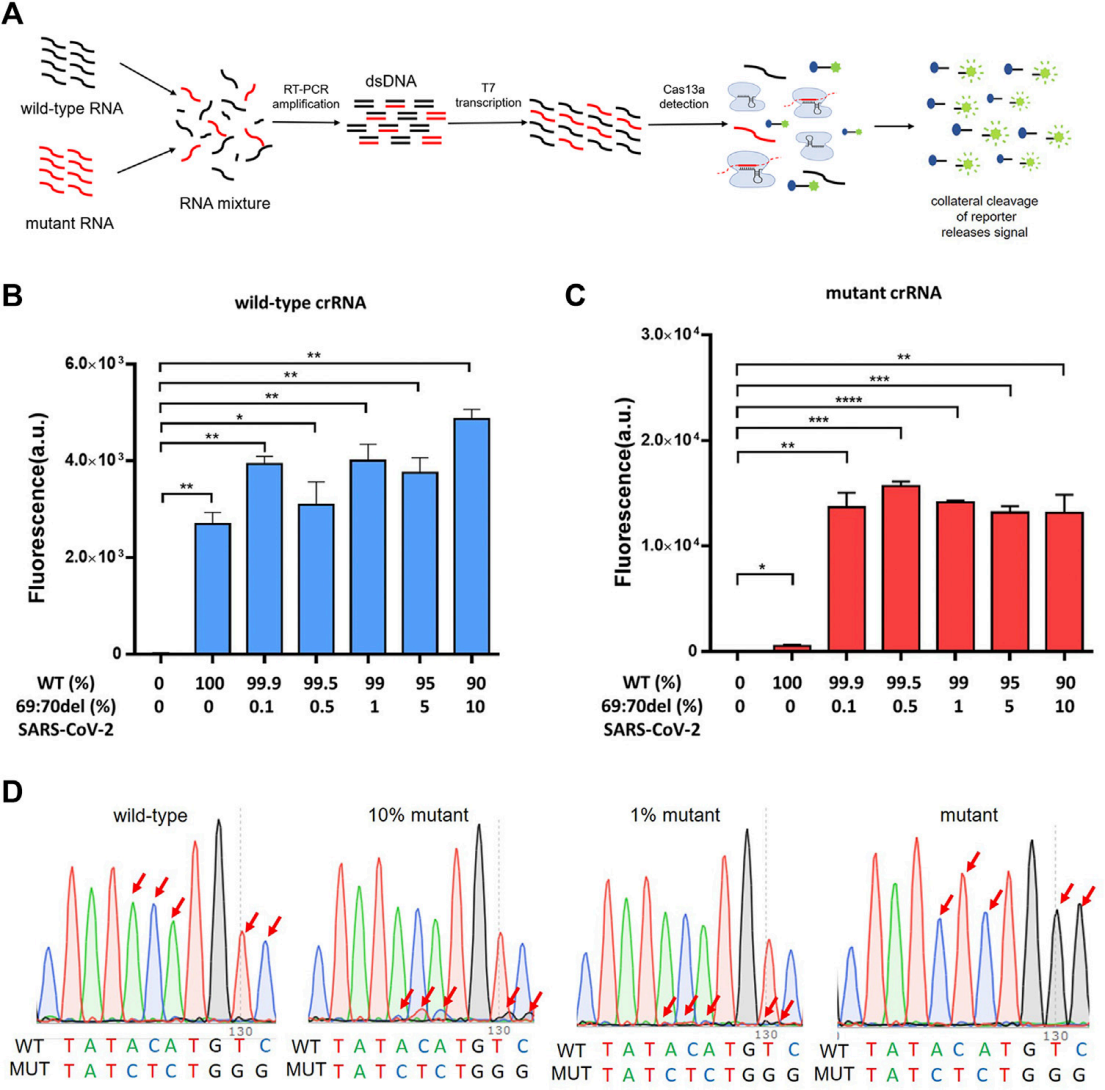
The PCR-CRISPR method detects mixed mutant genes of low proportion. **(A)** Schematic of PCR-CRISPR detection of mutant RNA on a background of wild-type RNA. **(B)** PCR-CRISPR detects mixed wild-type RNA of different proportions on a background of RNA mixture. **(C)** PCR-CRISPR detects mixed mutant RNA of different proportions on a background of RNA mixture. **(D)** Sequencing peak of Sanger sequencing for RNA mixtures of different proportions. (*n* = 3 technical replicates, two-tailed Student t-test; **, *p* < 0.01; ***, *p* < 0.001; ****, *p* < 0.0001; ns, no significant difference; bars represent mean ± s.e.m.).

To verify that the PCR-CRISPR method was capable of efficiently detecting low-proportion mutant genes in mixed samples, we tested different proportions of mixed samples (10%, 5%, 1%, 0.5%, and 0.1%) and compared the results with those of Sanger sequencing (Figure 3A). The results showed that PCR-CRISPR testing could detect mixed samples as low as 0.1% (Figures 3B,C), while the gene sequencing technology results showed that it could only distinguish between ≥10% of mixed mutant genes (Figure 3D). Thus, it indicates that PCR-CRISPR method could detect a low proportion of target nucleic acids in complex nucleic acid samples.

### Combination of PCR-CRISPR With ERASE Strip to Detect SARS-CoV-2 Variants

**FIGURE 4:**
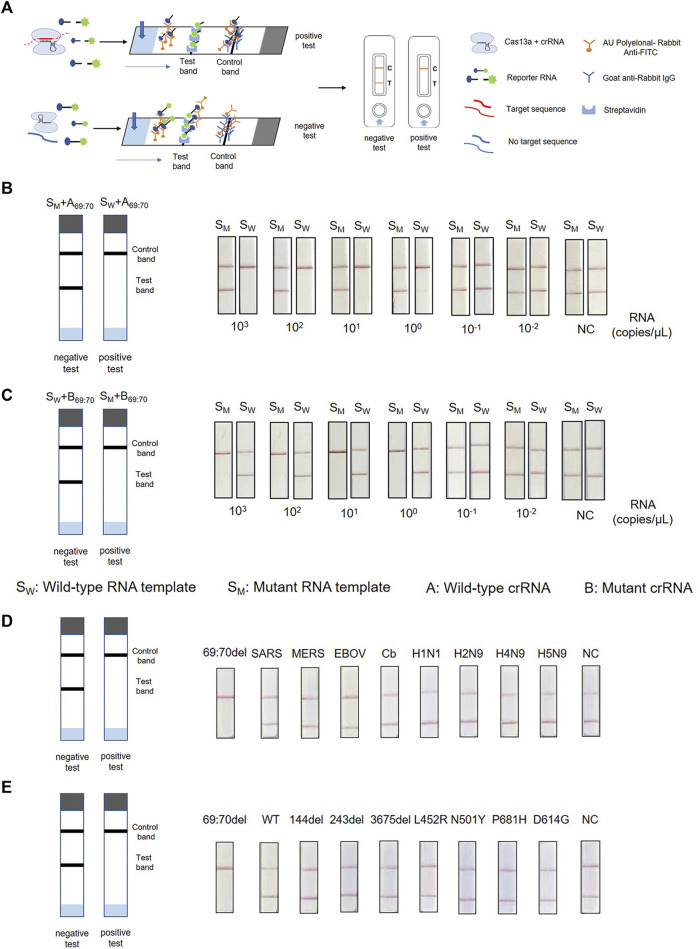
Combination of PCR-CRISPR detection with ERASE strip for lateral flow readout. **(A)** Schematic of PCR-CRISPR detection with lateral flow strip. **(B,C)** Sensitivity of PCR-CRISPR detection with lateral flow strip. **(B)** Detection of wild-type target RNA by PCR-CRISPR and wild-type crRNA followed by application to the lateral flow strip. **(C)** Detection of mutant target RNA by PCR-CRISPR and mutant crRNA followed by application to the lateral flow strip. **(D,E)** Specificity of PCR-CRISPR detection with the lateral flow strip. **(D)** PCR-CRISPR with lateral flow strip can discriminate other viral RNA. **(E)** PCR-CRISPR with lateral flow strip achieves specific detection of other viral mutant or deletion sites.

To achieve on-site testing of the SARS-CoV-2 variants, we combined PCR-CRISPR detection with lateral flow strip—ERASE. After the CRISPR reaction completed, the reaction products are fully added onto ERASE strip and the test results could be judged by naked eyes. To determine the sensitivity of this assay, RNA templates were prepared using a 10-fold gradient dilution and then detected using strip-based PCR-CRISPR (Figure 4A). As shown in Figures 4B,C, the sensitivity detection results are consistent with the fluorescence detection results and can reach 1–10 copies/μL. To evaluate the specificity of ERASE strip detection, we used nucleic acids from ten pathogens and different mutant site nucleic acids for the strip-based PCR-CRISPR test. It shows that as the same with the fluorescence-based PCR-CRISPR assay, the strip-based PCR-CRISPR assay could specifically distinguish the SARS-CoV-2 containing HV69-70del mutation with other pathogens (Figures 4D,E). Simultaneously, to show its practicability for identifying SARS-CoV-2 variants, we had also detected other common variants, such as N501Y, D614G, and P681H using this strip-based PCR-CRISPR assay (Supplementary Figure S3).

## Discussion

Continued efforts are being made toward global COVID-19 pandemic prevention and control, however, SARS-CoV-2 variants continue to emerge with mutations at different sites (Yaniv et al., 2021). As defined by the World Health Organization, five variants of concern (VOC) and six variants of interest (VOI) have emerged since the beginning of the SARS-CoV-2 pandemic (Duchene et al., 2020). The increased transmissibility of certain SARS-CoV-2 variants poses serious challenges to the prevention and control of the pandemic. Amino acid mutation sites, such as N501Y, E484K, and HV69-70del cross-appear or appear simultaneously in multiple VOC and VOI variants, indicating that SARS-CoV-2 has undergone adaptive changes and evolution during the COVID-19 pandemic in the process of continuous adaptation to the host (Li Q. et al., 2021; Kemp et al., 2021; Lubinski et al., 2022). In this study, we chose HV69-70del mutation site as the detect target to identify the Alpha and Omicron variants. Besides, based on PCR-CRISPR method, we continually developed the detection method for N501Y, D614G, and P681H mutant sites of SARS-CoV-2. It indicates its practicability of PCR-CRISPR method in identifying various SARS-CoV-2 variants.

Currently, main national health authorities are employing gene sequencing of patients’ samples to identify SARS-CoV-2 variants, however, this method is expensive and time-consuming (McNamara et al., 2020; Meredith et al., 2020; Bhoyar et al., 2021). Furthermore, when outbreaks occur locally and spread quickly, gene sequencing will not meet the needs of large-scale rapid screening. Presently, several countries and regions have carried out mixed testing of unknown SARS-CoV-2 samples to achieve the goal of rapid screening of positive cases (Bogere et al., 2021; Heaney et al., 2021; Hofman et al., 2021), which requires a higher sensitivity of testing methods. Our experiments showed that the PCR-CRISPR method could detect 0.1% of target nucleic acids in mixed samples (Wen et al., 2021), providing new insights for large-scale rapid screening of variants (Figure 3C). Certainly, this method cannot replace gene sequencing, but it has the ability to quickly detect clusters and help guide gene sequencing, making it an invaluable tool in the SARS-CoV-2 testing toolbelt. Furthermore, the method described in this study can be extended to the rapid detection of other pathogen variants (Kumar et al., 2021).

By combining the inherent high sensitivity and specificity of the PCR-CRISPR system with the simplicity of the ERASE lateral flow strip, we could read the results by naked eyes without fluorescence equipment. Compared with previously reported CRISPR-based SARS-CoV-2 variant detection methods (Zhang W. S. et al., 2021), ours provides a practical on-site test method to detect SARS-CoV-2 HV69-70del with obvious advantages. For example, strip-based PCR-CRISPR assay could eliminate the need for expensive fluorescence detectors, and improve the ability to diagnose SARS-CoV-2 variant on-site, particularly in under-developed and resource-limited regions (Myers et al., 2013; Li H. et al., 2021). Besides, by combined with smartphone-assisted visualization tools (Wen et al., 2021) or more accurate isothermal amplification, CRISPR detection system is expected to become a robust, rapid, quantitative and field-deployable POCT method for SARS-CoV-2 variants.

## Conclusion

As SARS-CoV-2 variants continue to evolve, identifying variants and rapidly adapting diagnostics to track variants will be critical to containing the ongoing COVID-19 pandemic (de Puig et al., 2021). In this study, we report a PCR-CRISPR method for SARS-CoV-2 HV69-70del mutant site detection with high specificality and sensitivity. By combined with the ERASE strip, it could enable rapid detection in resource-limited regions without fluorescence equipment, which provide a powerful tool for SARS-CoV-2 variants early identification.

## Data Availability

The original contributions presented in the study are included in the article/Supplementary Material, further inquiries can be directed to the corresponding authors.
