# Supplementary material for: Highly Sensitive Detection Method for HV69-70del in SARS-CoV-2 Alpha and Omicron Variants Based on CRISPR/Cas13a
Source: Front Bioeng Biotechnol. 2022 Apr 12;10:831332. doi: 10.3389/fbioe.2022.831332 (PMC9039052; doi:10.3389/fbioe.2022.831332)
Supplement: Supplementary file 1 [file DataSheet1.docx]

***Supplementary Material***

1. **Supplementary Figures and Tables**
   1. **Supplementary Figures**

**Figure S1.** Time analysis of wild-type and mutant RNA dilution series with RT-PCR+CRISPR.

**
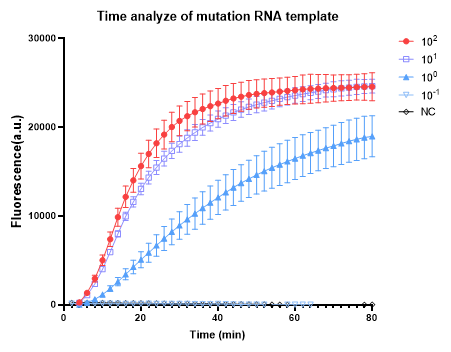
**

**Figure S2. (A)** PCR+CRISPR can discriminate different viral mutant or deletion sites. **(B)** PCR+CRISPR achieves sensitive and specific detection of different viral RNA. (n=3 technical replicates, two-tailed Student t-test; ***, p < 0.001; ****, p < 0.0001; bars represent mean ± s.e.m.).

**
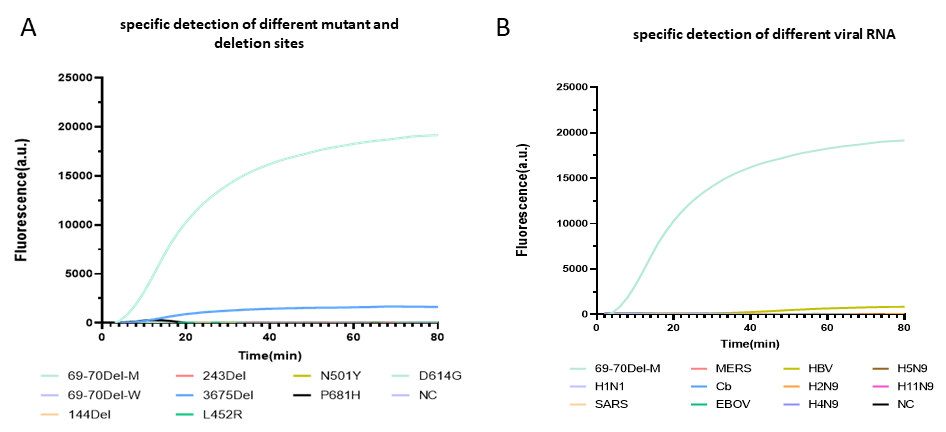
**

**Figure S3.** RT-PCR+CRISPR with lateral flow strip was used to detect N501Y, P681H, and D614G mutation.
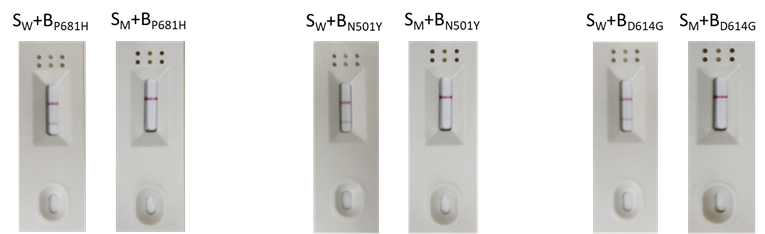


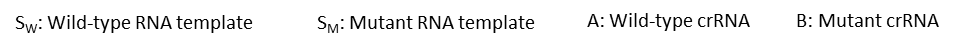


- 1. **Supplementary Tables**

**Supplementary Table 1** The primers used in this study.

| **Name** | **Sequences (5’-3’)** |
| --- | --- |
| 6970-Primer 1-F | AATTCTAATACGACTCACTATAGGGAGAACTCAATTACCCCCTGC |
| 6970-Primer 2-F | AATTCTAATACGACTCACTATAGGGAGAACTCAATTACCCCCTGCA |
| 6970-Primer 3-F | AATTCTAATACGACTCACTATAGGGTCAATTACCCCCTGCATACACT |
| 6970-Primer 1-R | TGGTAGGACAGGGTTATCAA |
| 6970-Primer 2-R | CCCAGAGATAGCATGGAACCA |
| 6970-Primer 3-R | TGGTCCCAGAGATAGCATGGA |
| 501-P1-F | AATTCTAATACGACTCACTATAGGGTCAGGCCGGTAGCACACCTT |
| 501-P2-F | AATTCTAATACGACTCACTATAGGGCCGGTAGCACACCTTGTAAT |
| 501-P1-R | AGAAGTTCAAAAGAAAGTACT |
| 501-P2-R | TGTAGAAGTTCAAAAGAAAGT |
| 681-P1-F | AATTCTAATACGACTCACTATAGGGCAACAACTCATATGAGTGTGA |
| 681-P2-F | AATTCTAATACGACTCACTATAGGGAACTCATATGAGTGTGACATAC |
| 681-P1-R | AGCAACTGAATTTTCTGCAC |
| 681-P2-R | ACTGAATTTTCTGCACCAAGT |
| 614-P1-F | AATTCTAATACGACTCACTATAGGGTTTGGTGGTGTCAGTGTTAT |
| 614-P2-F | AATTCTAATACGACTCACTATAGGGGGTGGTGTCAGTGTTATAA |
| 614-P1-R | AGTAGGAGTAAGTTGATCTG |
| 614-P2-R | TAGGAGTAAGTTGATCTGCA |

**Supplementary Table 2** The crRNA sequences used in this study.

| **Name** | | **Sequences (5’-3’)** |
| --- | --- | --- |
| T7-crRNA-F | | taatacgactcactatagGGGATTTAGACTACCCCAA |
| Mut6970-crRNA1 | GGGATTTAGACTACCCCAAAAACGAAGGGGACTAAAACccattggtcccagagatagcatggaacc | |
| Mut6970-crRNA2 | GGGATTTAGACTACCCCAAAAACGAAGGGGACTAAAACttggtcccagagatagcatggaaccaag | |
| Mut6970-crRNA3 | GGGATTTAGACTACCCCAAAAACGAAGGGGACTAAAACgtcccagagatagcatggaaccaagtaa | |
| Mut6970-crRNA4 | GGGATTTAGACTACCCCAAAAACGAAGGGGACTAAAACccagagatagcatggaaccaagtaacat | |
| Mut6970-crRNA5 | GGGATTTAGACTACCCCAAAAACGAAGGGGACTAAAACgtaccattggtcccagagatagcatgga | |
| Wt6970-crRNA1 | GGGATTTAGACTACCCCAAAAACGAAGGGGACTAAAACattggtcccagagacatgtatagcatgg | |
| Wt6970-crRNA2 | GGGATTTAGACTACCCCAAAAACGAAGGGGACTAAAACtggtcccagagacatgtatagcatggaa | |
| Wt6970-crRNA3 | GGGATTTAGACTACCCCAAAAACGAAGGGGACTAAAACggtcccagagacatgtatagcatggaac | |
| Wt6970-crRNA4 | GGGATTTAGACTACCCCAAAAACGAAGGGGACTAAAACgtcccagagacatgtatagcatggaacc | |
| Mut501-crRNA1 | GGGATTTAGACTACCCCAAAAACGAAGGGGACTAAAACggtaaccaacaccatAagtgggttggaa | |
| Mut501-crRNA2 | GGGATTTAGACTACCCCAAAAACGAAGGGGACTAAAACaaccaacaccatAagtgggttggaaacc | |
| Wt501-crRNA1 | GGGATTTAGACTACCCCAAAAACGAAGGGGACTAAAACggtaaccaacaccattagtgggttggaa | |
| Wt501-crRNA2 | GGGATTTAGACTACCCCAAAAACGAAGGGGACTAAAACaaccaacaccattagtgggttggaaacc | |
| Mut681-crRNA1 | GGGATTTAGACTACCCCAAAAACGAAGGGGACTAAAACcgtgcccgccgaTgagaattagtctgag | |
| Wt681-crRNA1 | GGGATTTAGACTACCCCAAAAACGAAGGGGACTAAAACcgtgcccgccgaggagaattagtctgag | |
| Mut614-crRNA1 | GGGATTTAGACTACCCCAAAAACGAAGGGGACTAAAACctgtgcagttaacaccctgataaagaac | |
| Mut614-crRNA2 | GGGATTTAGACTACCCCAAAAACGAAGGGGACTAAAACttctgtgcagttaacaccctgataaaga | |
| Wt614-crRNA1 | GGGATTTAGACTACCCCAAAAACGAAGGGGACTAAAACctgtgcagttaacatcctgataaagaac | |
| Wt614-crRNA2 | GGGATTTAGACTACCCCAAAAACGAAGGGGACTAAAACttctgtgcagttaacatcctgataaaga | |

**Supplementary Table 3** The report RNA sequences used in this study.

| **Report RNA** |  |
| --- | --- |
| FB-polyU-14nt | FAM-UUUUUUUUUUUUUU-BIO |
| FB-polyU-20nt | FAM-UUUUUUUUUUUUUUUUUUU-BIO |

**Supplementary Table 4** The template sequences used in this study.

| **Mutation site** | | **Sequences (5’-3’)** |
| --- | --- | --- |
| Wt6970 | gatcctcagttttacattcaactcaggacttgttcttacctttcttttccaatgttacttggttccatgctatacatgtctctgggaccaatggtactaagaggtttgataaccctgtcctaccatttaatgatggtgtt | |
| Mut6970 | GatcctcagttttacattcaactcaggacttgttcttacctttcttttccaatgttacttggttccatgctaTctctgggaccaatggtactaagaggtttgataaccctgtcctaccatttaatgatggtgtt | |
| Wt501 | tcaggccggtagcacaccttgtaatggtgttgaaggttttaattgttactttcctttacaatcatatggtttccaacccactAatggtgttggttaccaaccatacagagtagtagtactttcttttgaacttctaca | |
| Mut501 | tcaggccggtagcacaccttgtaatggtgttgaaggttttaattgttactttcctttacaatcatatggtttccaacccactTatggtgttggttaccaaccatacagagtagtagtactttcttttgaacttctaca | |
| Wt681 | caacaactcatatgagtgtgacatacccattggtgcaggtatatgcgctagttatcagactcagactaattctcCtcggcgggcacgtagtgtagctagtcaatccatcattgcctacactatgtcacttggtgcagaaaattcagttgct | |
| Mut681 | caacaactcatatgagtgtgacatacccattggtgcaggtatatgcgctagttatcagactcagactaattctcAtcggcgggcacgtagtgtagctagtcaatccatcattgcctacactatgtcacttggtgcagaaaattcagttgct | |
| Wt614 | tttggtggtgtcagtgttataacaccaggaacaaatacttctaaccaggttgctgttctttatcaggAtgttaactgcacagaagtccctgttgctattcatgcagatcaacttactcctact | |
| Mut614 | tttggtggtgtcagtgttataacaccaggaacaaatacttctaaccaggttgctgttctttatcaggGtgttaactgcacagaagtccctgttgctattcatgcagatcaacttactcctact | |
